# Promotion of Healthy Eating in Spanish High Schools

**DOI:** 10.3390/nu12071979

**Published:** 2020-07-03

**Authors:** Almudena Garrido-Fernández, Francisca María García-Padilla, José Luis Sánchez-Ramos, Juan Gómez-Salgado, Juan Diego Ramos-Pichardo, Ángela María Ortega-Galán

**Affiliations:** 1 Department of Nursing, Faculty of Nursing, University of Huelva, 21071 Huelva, Spain; almudena.garrido@denf.uhu.es (A.G.-F.); fmgarcia@denf.uhu.es (F.M.G.-P.); jsanchez@denf.uhu.es (J.L.S.-R.); juan.ramos@denf.uhu.es (J.D.R.-P.); angela.ortega@denf.uhu.es (Á.M.O.-G.); 2 Department of Sociology, Social Work and Public Health, Faculty of Labour Sciences, University of Huelva, 21007 Huelva, Spain; 3 Safety and Health Postgraduate Programme, Universidad Espíritu Santo, Guayaquil 092301, Ecuador

**Keywords:** adolescents, diet, food and nutrition, health promotion, schools, students

## Abstract

Obesity and overweight are both public health problems, affecting increasingly younger populations. Promoting healthy eating should be a must in schools. Therefore, getting to know the eating habits of a population group as sensitive as adolescents and whether their schools apply an appropriate policy for their nutrition should be a priority. Therefore, the objective of this study was to discover whether the Secondary Education High Schools of Huelva and its province could be considered as centres that promote a healthy diet. A cross-sectional descriptive study was developed using a questionnaire comprising 39 indicators that were evaluated on 5 previously piloted subcategories of validated information. Data were obtained through a questionnaire presented to 200 key informants with four different profiles. The highest score for promoting healthy eating in the centres was related to the subcategory “School Curriculum”, whereas the lowest means were those related to the Community category. No practical activities such as outings or cooking workshops were carried out. The low participation in activities promoting healthy eating habits, research and health training must be highlighted. Little attention was paid to compliance and monitoring of school cafeterias. Most of the studied Secondary Education High Schools did not meet the requisites to be considered promoters of healthy eating habits. Only three of the studied centres can be considered healthy-eating promoters. Institutional commitment is needed to favour the intersectorality of the different agents implied and to provide teaching units and other teaching profiles with the necessary resources, training, and tools to achieve integral and protective teaching activities aimed at promoting students’ healthy eating habits.

## 1. Introduction

Nutrition is one of the most relevant aspects of childhood and adolescent care due to its implications for a person’s integral development [1].

Obesity and overweight, and in general bad eating habits, have virtually increased epidemically in the last two decades, concerning increasingly younger individuals and making interventions in this regard necessary [2,3,4,5,6]. The World Health Organization (WHO) has listed childhood obesity as “one of the most serious public health problems of the 21st century, progressively affecting low- and middle-income countries and, above all, urban areas” [7]. In 2016, 340 million children and adolescents (aged 5 to 19 years) were classified as overweight or obese [8].

The World Health Organization recommends arranging school policies and programmes that promote healthy eating and implementing specific actions to teach nutrition and culinary skills in schools, considering also necessary to incorporate families and caregivers [9]. A community approach is therefore essential, where intersectorality, participation and orientation to health assets are enhanced [10,11]. Different experiences highlight the effectiveness of this approach [11,12].

The transition from school to high school can promote new habits in adolescents as, at this age, they often have more access to and choose more autonomously unhealthy food options, thus limiting the influence of parents on their eating behaviour [4,13]. The school timetable for Secondary Education High Schools (SEHS) of Huelva is usually from 8:30 a.m. to 3:30 p.m., with half an hour of break. In this break, students consume some food, usually a sandwich. The centres do not provide food to students, but students eat what they have brought from home or buy something in the existing school cafeterias or in nearby stores [14].

School breakfast is particularly important for its influence on the daily nutritional balance and its contribution to improving school performance [15,16,17,18,19,20]. Most adolescents either skip breakfast or have an unhealthy or inappropriate one [6,14,21]. Thus, educational strategies promoting healthy eating and for this age group are of key relevance, as habits acquired in childhood and adolescence are usually maintained in adulthood [22,23,24,25,26].

Nutritional health seems to hold an unimportant place in the high school curriculum, differently from earlier stages of education such as primary and/or child education. Food-related health protection is insufficient: food in school cafeterias has poor quality, external premises promote the consumption of unhealthy food, and health-promoting activities are lacking [27,28,29].

Traditionally, health-related issues [30] have been considered a responsibility of the health field in Spain. However, numerous studies highlight the importance of the education sector in this field [31,32]. The role of the educational community in food and nutrition education in Primary and Child Education is identified, recognising the importance that teachers, the Administration Board and the School Centres have in this area, as well as the inclusion of teaching topics or programmes for the promotion of healthy habits [33,34,35].

Teacher training in this field [30,33] is essential, as deficiencies have been identified regarding food-related subjects at the initial training levels [36,37]. Nutritional education in the school environment and educational interventions improve the general diet of young people, breakfast in particular, and provide greater food safety [3,4,14] as well as improvements in reducing obesity [32] or increasing the consumption of fruits and vegetables [38].

Promoting healthy eating should be a must in schools; therefore, the main objective of this research is to discover whether Secondary Education High Schools of Huelva and its province can be considered healthy-eating promoters.

## 2. Materials and Methods

### 2.1. Design

A cross-sectional descriptive study was carried out, taking public SEHS of the province of Huelva (Spain) as the unit of analysis.

### 2.2. Participants

Of the 60 public SEHS in the province, a total of 22 SEHS and 200 subjects participated in the study. Professionals from different areas of the SEHS were selected:

- Management Teams and Counsellors
- Tutor and non-tutor teachers of subjects that included the topic of food in their curriculum, such as Biology, Physical Education and certain subjects included in Vocational Training Cycles.

In addition, the following data were collected: sex, age, years of teaching, educational level taught, subject taught, teaching post and management post of each participant.

In order to record information on the indicators related to Community participation and Training of other school community members, Parents’ Associations leaders of the study centres were surveyed.

### 2.3. Variables and Data Collection Technique

Indicators used for the assessment of SEHS as health-eating promoters were extracted from a document validated through a Delphi study [39]. In this research, a total of 53 Spanish experts of different disciplines (nurses, physicians, pharmacists, veterinarians, nutritionists, health promotion technicians, high school and university teaching staff involved in health and education areas and family members of high school students who were part of the Parents’ Associations) participated, from both the health and the education sectors, reaching a consensus on 56 indicators, divided into 3 categories (Table 1).

This research focused on indicators related to the Centre and the Community, not studied so far. Aspects related to school cafeterias were analysed in an Andalusian-related study, whose results will be extrapolated to our context [27].

### 2.4. Preparation of the Instrument and Information Collection

Direct questions were asked on all the indicators for their measurement through a self-administered survey. These were assessed by judges through a test and piloted on a sample of 10 teachers. After piloting, some questions were removed, and the wording of others was modified, eventually resulting in a questionnaire with 57 questions distributed in study subcategories as follows: Centre Educational Project (17 Questions), School Curriculum (25 Questions), Teacher Training (9 Questions), Community Engagement (5 Questions), Training of other School Community Members (1 question).

Questions about the indicators under study were incorporated into four different questionnaires depending on the four profiles of informants that could best answer them. All the informants valued the actions that were being carried out in the centre for the promotion of healthy eating on a scale from 1 to 3, being (1) Never or Almost Never, (2) Sometimes, and (3) Always or Almost Always.

Also, an open question dedicated to activities promoting healthy eating performed at the centres was included and manually categorised.

To request access to the study centres, the Provincial Delegation of Education was contacted. Subsequently, visits to the SEHS were arranged with the management teams by telephone and email.

Once in the centre, the project was explained to the management team and/or counsellors. If they agreed to participate, they were asked for information on two topics:

- The existence or not of a cafeteria in the SEHS
- The number of teachers instructing in subjects linked to the study subject and if eating habits were included in the Tutorial Action Plan. All this was done to calculate the number of questionnaires to be delivered to each centre.

The management team person contacted was in charge of delivering the questionnaires to the different professionals, always stating the voluntary participation, so no incentives were given to the participants.

Additionally, a telephone survey was performed involving Parents’ Associations leaders, with 13 multiple-choice closed questions which assessed indicators on Community Engagement and Training of other school community members. Of the 22 centres, 17 had active Parents’ Associations, and contact with them was made through the centre (5 Parents’ Associations) or by telephone or mail (12 Parents’ Associations).

### 2.5. Data Analysis

The data analysis was carried out using the SPSS statistics version 25 licensed by the University of Huelva. The overall mean and the mean per subcategory were obtained from each participating centre. Means were also obtained by indicator and subcategory in general. The theoretical median of possible values obtained for each question of the questionnaire would be 2. The indicator was met when a score above 2 was obtained, and it was not met when the score of the question was less than 1.50.

Data on healthy-eating promotion activities were manually categorised.

### 2.6. Ethical Considerations

This study was approved by the ethics committee of the University of Huelva and obtained the Authorisation of the Delegation of Education of Huelva. The Ministry of Education and Sport also established a collaboration agreement with the University of Huelva for the development of interventions in non-university public teaching centres of the Ministry of Education and Sport.

The application of Organic Law 3/2018 of December 5 on the Protection of Personal Data and Guarantee of Digital Rights was guaranteed, not using such data for purposes other than those set out in the objectives and specific purposes of this study [40]. In addition, the study was conducted following ethical principles set out in the Helsinki Declaration [41].

Participation in the study was free and voluntary. An informed consent model was delivered to the participants, ensuring the confidentiality of the data and their exclusive use for the aim of the study.

## 3. Results

For the 371 questionnaires submitted, the total response rate was 53.9% (n = 200).

In relation to the response rate, 90.9% of management teams’ members, 77.3% of counsellors, 86.4% of non-tutors and 59.1% of tutors answered the questionnaire.

The identifying characteristics of the participants are shown in Table 2.

As for the assessment of the centres as healthy-eating promoters, an overall mean of 1.79 was obtained (SD = 0.16). Those actions promoting nutritional health in which the centres obtained a high score were related to the subcategory 2 “School Curriculum” (1.95 SD = 0.15), whereas those related to the Community category obtained the lowest means (Table 3).

The global means per centre ranged from 2.15 to 1.51. Only 3 centres of the 22 studied SEHS obtained an overall mean above 2 (Table 4); therefore, it can be said that only 13.6% of the studied centres can be considered healthy-eating promoters.

Regarding the means by subcategories of each centre (Table 4), the following can be highlighted:

The School Curriculum subcategory obtained the best means in the centres; 45.45% of the centres (n = 10) exceeded the score of 2.

When assessing the Centre’s Educational Project subcategory, only six of the centres (27.27%) reached means above 2. Three subcategories showed the worst outcomes: Community Participation and School Community Training, for which 40.90% of SEHS (9) did not meet the expected mean, and Teacher Training, for which no centre reached the average of 2.

Above each category and study subcategory (Table 5), indicators that reached the highest scores include the following characteristics:

*In the Centre’s Educational Project subcategory:*

The centre’s Tutorial Action Plan includes healthy eating actions for students (2.63).

*In the School Curriculum subcategory:*

When I work on healthy eating habits in class, these are related to physical activity (2.90).

The lowest scores in Table 5 are related with the following indicators:

*From the Centre’s Educational Project subcategory:*

There are records on incidents of non-compliance with regulations regarding the existing food supply in the school cafeteria (1.08).

*From the School Curriculum subcategory:*

If practical activities are carried out, some are related to organising outings, taking advantage of the food topic: visits to the fruit shop, butcher’s shop, bakery, etc. (1.24).

*From the Teacher Training subcategory:*

There is a health promotion line working on school research and innovation (1.29).

*From the Community Participation subcategory:*

Cooking workshops are held with community and cooperative participation (1.27).

*From the Community Formation subcategory:*

Healthy-eating actions are carried out for the cafeteria staff (1.35).

As for the practical activities on healthy eating carried out in the centres, obtained from an open question, the most frequent were: the “Healthy or Andalusian Breakfast Day”, consisting of fruit and bread with oil; talks on healthy eating by experts; tasks dealing with anthropometric measurements, assessment of fruit consumption or games by Physical Education teachers; extra points for consuming fruit or nuts after physical activity; making posters (analysis of sugar consumption in normal drinks, healthy breakfast, etc.), nutrients calculations, interpretation of labelling or testing of daily consumption made by biology teachers; food preparation or food competitions.

Regarding the data collected from the Parents’ Associations, only three responses were provided. Of the 13 questions asked related to community activities and training, only 2 items scored positively: the interest of Parent’s Associations in participating in health eating formative and promotional programs and the participation of Parents’ Associations in school activities. The representatives of these Parents’ Associations stated that healthy food consumption is only sometimes encouraged by the school centres, as are healthy-eating promotion activities. The rest of the variables were perceived in an unflattering way: after-school activities do not encourage healthy food consumption neither they include community-involved cooking workshops; families do not receive any documentation on healthy eating information from the school centre and do not participate in food promotion activities at the school; families never or almost never collaborate with Parents’ Associations on training and nutrition activities.

## 4. Discussion

This research aimed at assessing high schools’ degree of implication in the promotion of healthy eating habits among adolescents. Although the response rate was lower than expected, an assessment of all indicators in the 22 studied centres was achieved. “No Answer” occurred mainly in the collective of Tutor Teachers. Initially, the questionnaires were delivered to the tutors of all courses, although the content on healthy eating promotion integrated in the Tutorial Action Plan was only addressed to participants involved in some educational levels, usually the third course of Compulsory Secondary Education, which amounted for most respondents of our study. In addition, this lack of response was justified, on behalf of the centres, by the workload that tutors had in the centres.

Regarding the results obtained, it is noted that the promotion of healthy eating is not a priority in the SEHSs of the province of Huelva, beyond the curricular compliance established in the current educational legislation [42]; this may be either because healthy eating training is more commonly associated with Primary Education than with Secondary Education [33,34,35] or because families are understood to be the main food providers for adolescent students. Although there is a concern to offer a food service, either in a cafeteria or in a dining room, to help achieve a work–life balance, there is no interest in the quality of food at school or in improving students’ eating habits. Therefore, there is a need to improve the nutritional quality of foods available in school canteens [43].

Those indicators with a lower score are related to the control of the centres over cafeteria service. This is consistent with the transfer of cafeterias’ food supply responsibility to those in charge [27] of the service, as they are not monitored by any external authority to ensure compliance with the current legislation [44]. It is equally important to note that SEHS with no cafeteria service were among the ones which most greatly promoted healthy eating; of the five SEHSs under study without a school cafeteria, three obtained the highest scores. This may be because they assumed a commitment to ensuring healthy eating habits during the school day, while other centres delegated this responsibility to cafeterias. These results are highly in line with the idea of the school cafeteria as a negative predictor of healthy eating during the school day [14,28].

School Curriculum was expected to be the best assessed category, as it is determined by the legislation that regulates mandatory actions such as the contents established for the curricula of subjects like Physical Education, Biology or Natural Sciences [42] or those established in the Tutorial Counselling and Action Plans [45] in which Healthy Eating appears as one of the topics to be addressed. It is true that the promotion of healthy eating appears in tutorial action plans and, therefore, is included in the centres’ educational projects, but no innovative teaching activities were observed that consider the integration and continuity of healthy-eating promotion within the school academic curriculum. Activities focused on promoting or influencing the attitude of students towards healthy eating were essentially generated by Physical Education teachers, who appeared to be the main Health Assets, among other reasons, for being convinced of the benefits of these habits if they are incorporated into our lifestyles [2,12,46,47].

Among the most deficiently scored subcategories, we highlighted Teacher Training in promoting healthy eating. It seems that this collective does not feel the need for training in this area. This collective does not claim hetero-training nor auto-training because they do not consider the need to address this issue professionally. Tutors who may have this need do not demand training, because their interventions within the Tutorial Action Plan are given by the centre counsellor. Other studies show teachers’ ignorance of existing resources and lack of training in this area, which is an important limitation when promoting healthy eating habits [33,34,35]. This, together with the lack of sound policies, leads to non-committal attitudes towards these interventions within the educational field.

The participation of the family in the educational field, in this or any other, is indisputable today [48], as well as the need for coordination with other community institutions or agents to achieve effectiveness in educational interventions [49,50]. However, in the studied context, we found lack of participation of members of the community such as the parents of students in actions to promote healthy eating, except in one-time activities of commemorative or festive nature. The lack of contribution of these educational agents may have its origin in the rather aesthetic consideration of their presence in the interests of the democratisation of schools or in the low participatory culture of families through Parents’ Associations for greater involvement (in social terms, political rhetoric highlights and fosters parents’ participation, while this does not seem to be actually implemented nor even considered or favoured). In our study, a poor response from Parents’ Associations was proved, which strengthens the hypothesis of little interest or time for the promotion of adolescents’ healthy eating in the school environment.

The data in this study are self-declared. As a weakness, the low response rate may be highlighted, especially unexpected in the case of Parents’ Associations.

Among the strengths, the representative capacity of the sample for the region of Huelva and the scarcity of studies on the promotion of healthy eating that consider all members of the educational community is remarkable. Thus, this study may constitute a novelty to encourage future actions.

As future lines of intervention, the creation of a global model that involves the different actors (social, educational, family and health) is proposed. This may be done by carrying out interventions that provide teachers and different educational profiles with the necessary resources, training and tools to carry out a comprehensive and protective teaching activity aimed at promoting students’ healthy eating habits.

## 5. Conclusions

Most centres appeared to be below the established mean corresponding to healthy eating promoters; only three of the centres studied can be considered as such.

The School Curriculum subcategory obtained the best mean, both globally and by centres. The most negative results were obtained in indicators related to the Community category.

In the analysis of each study indicator, a large number of indicators did not reach the expected scores for the promotion of healthy eating. Bibliographic resources available at the school library were not usually used, and coordination with the educational team regarding this topic seemed to be lacking. Also, the lack of participation in food and health training activities and in related lines of research is highlighted. Community participation was not much considered, and no attention was paid to regulation compliance and monitoring in school cafeterias.

In contrast, the results indicate that the centres’ tutorial action plan and the educational project envisaged actions to promote healthy eating. The centres had educational resources on healthy eating and included health and food content in a cross-sectional way. The most commonly used resource on healthy eating within the classroom environment was the food pyramid.

Practical activities on healthy eating in the centres were usually one-time, which resulted in the generally scarce promotion of healthy eating habits fostered by secondary education high schools. This fact justifies the need to join forces towards an intersectoral approach to achieve a real improvement in students’ healthy eating habits.

## Figures and Tables

**Table 1:** Indicators of healthy-eating promotion.

Categories	Subcategories	No of Indicators
**Centre**	Centre’s Educational Project	15
	School Curriculum	7
	Teacher Training	6
**Community**	Community participation	4
	Training of other school community members	7
**School cafeteria**	Food options	9
	Location of healthy products	2
	Publicity on healthy products	6
**Total**		56

**Table 2:** Frequency of identifying characteristics of the informants.

		N	Frequency (%)
**Profile**	Management team	62	31
	Counsellors	17	8.5
	Non-tutors	75	37.5
	Tutors	46	23
**Sex**	Male	85	42.5
	Female	115	57.5
**Age**	30–40	61	30.5
	20–30	5	2.5
	40–50	70	35
	50–60	59	29.5
	60	5	2.5
**Years teaching**	>10 years	151	75.5
	<10 years	49	24.5
**Level of education**	Higher secondary	12	6
	Secondary	99	49.5
	Vocational training	9	4.5
	Various *	80	40
**Subject**	Physical education	29	14.5
	Health sciences	53	26.5
	Other	118	59

* Various: a single informant was teaching students at various educational levels, for example, both compulsory secondary education and higher secondary education.

**Table 3:** General global means and means per dimension.

	N	Minimum	Maximum	Mean	SD
**Global actions**	22	1.51	2.15	1.79	0.16
**CEP**	22	1.54	2.36	1.86	0.23
**SC**	22	1.68	2.29	1.95	0.15
**TT**	22	1.34	1.98	1.62	0.2
**CP**	21	1	2.5	1.59	0.37
**CT**	17	1	3	1.48	0.63

CEP = Centre’s Educational Project, SC = School Curriculum, TT = Teacher Training, CP = Community Participation, CT = Community Training.

**Table 4:** Mean Global Assessment of Secondary Education High Schools (SEHS) as healthy-eating promoters and by study categories.

		Global Mean	Mean	Mean	Mean	Mean	Mean
			(CEP)	(SC)	(TT)	(CP)	(CT)
**Centre**	1	1.81	1.73	2.02	1.71	1	1
	2	1.75	1.86	1.87	1.51	1.3	**2.5**
	3	1.74	1.6	1.87	1.78	1.6	1.67
	4	1.85	1.74	2.04	1.69	1.96	**2.25**
	5	1.67	1.87	1.76	1.34	1.51	1
	6	1.94	2.02	2.07	1.91	1.67	NA
	7	1.69	1.78	1.91	1.35	1.62	1
	8	1.51	1.59	1.73	1.34	1.35	1
	9	1.88	2.09	2.1	1.52	1.93	1.5
	10	1.6	1.72	1.78	1.41	1.27	1
	11	2.15	2.36	2.13	1.94	2.25	NA
	12	1.94	1.73	2.29	1.83	1.4	NA
	13	1.79	2.17	2.02	1.43	1.33	1
	14	1.7	1.67	1.92	1.67	1.37	1.75
	15	2.02	2.15	2.17	1.81	1.6	NA
	16	1.87	1.88	2.05	1.56	NA	NA
	17	1.6	1.54	1.89	1.56	1.18	1
	18	2.09	2.26	2.16	1.8	2.5	**3**
	19	1.8	1.71	1.83	1.7	1.7	**2**
	20	1.59	1.6	1.85	1.44	1.23	1
	21	1.67	1.94	1.68	1.39	2.1	1
	22	1.9	1.94	1.99	1.98	1.52	1.5

CEP = Centre’s Educational Project, SC = School Curriculum, TT = Teacher Training, CP = Community Participation, CT = Community Training; NA: no answers.

**Table 5:** Indicators of Healthy-Eating Promotion in SEHS. Profile of the informants of each indicator. Global means per indicator.

Indicators of Healthy Eating Promotion in SEHS/Profile of the Informants *	**MT**	**COUNSEL.**	**TEACHERS**	**TUTOR**	**Global Means**
**Centre’s Educational Project**					
1. Healthy nutrition and physical activity recommendations are made through different means of dissemination (posters, social networks, leaflets, radio, school newspaper…)	X	X			2.115
2. Students’ eating habits are assessed.	X	X			1.83
3. The centre’s educational Project includes actions on healthy eating habits aimed at students.	X	X			2.16
4. The centre’s educational Project includes actions on healthy eating habits aimed at teachers.	X				1.19
5. Recommendations are made for families and students on healthy food they could bring to be consumed in the centre.	X	X			1.88
6. If recommendations are made, compliance with food consumption in the centre is monitored.	X	X			1.64
7. Proposals for the inclusion of healthy products in the school cafeteria such as fruit, fresh tomato, olive oil, whole-grain products… are made.	X	X			1.94
8. There are specific regulations for the food options in the school cafeteria to be healthy, varied, balanced and adapted to the needs of the school community.	X	X			1.45
9. There are corrective measures in case of non-compliance with the regulations regarding the school cafeteria food options.	X	X			1.16
10. Compliance with existing regulations of the school cafeteria food options is monitored.	X	X			1.52
11. There are records of incidents related to non-compliance with the regulations regarding the school cafeteria food options.	X	X			1.08
12. The centre has didactic resources on healthy eating habits (library, departments…).	X	X	X		2.17
13. There is a coordinator for nutritional health and general health activities.	X	X			2.17
14. The centre’s Tutorial Action Plan includes actions on healthy eating habits aimed at students.		X			2.63
15. There are work groups of teachers to promote healthy eating habits in the centre (activities, curriculum integration, compliance with regulations monitoring…).	X	X			1.6
16. In my teaching centre, healthy eating is promoted.			X	X	1.85
17. The centre’s school project includes actions on healthy eating habits promotion.			X	X	2.06
**School curriculum**	**MT**	**COUNSEL.**	**TEACHERS**	**TUTOR**	**Global means**
1. The educational team is coordinated to approach health and eating habits issues.	X	X	X		1.69
2. The subjects in the school curriculum include aspects on the promotion of healthy eating habits stated by the Centre’s Educational Project.	X		X		**2.20**
3. There is a yearly calendar with activities aimed at training in healthy eating habits.	X	X			1.88
4. Textbooks and school supplies approach healthy eating habits.	X	X	X		2.09
5. Content on general health and healthy eating habits is cross-sectionally included as a project in departments’ programmes.	X	X			2.13
6 Further content on healthy eating habits is included in the school curriculum subjects, within the classroom context.	X	X			2.21
7. Practical activities for the promotion of healthy eating habits are carried out in the centre.	X	X	X	X	2
8. If there are practical activities, some are aimed at the preparation of food, juices, etc.	X	X	X	X	1.51
9. If there are practical activities, some deal with organising outings related to nutrition: visiting a greengrocer, butcher, bakery, etc.	X	X	X		1.24
10. Curriculum topics on healthy eating habits are included in social networks and digital platforms.	X	X			1.54
11. I find difficulty/limitations in promoting and implementing healthy eating practices.	X	X	X	X	1.73
12. The Tutorial Action Plan includes aspects on the promotion of healthy eating habits, included in the educational project.		X			2.65
13. Healthy eating activities are usually one-time: “Fruit Day”, “Andalusian Breakfast Day”, etc.			X	X	2.08
14. I design/adapt teaching units aimed at healthy eating and nutrition in my classroom context.			X		2.56
15. When dealing with nutrition in my lessons, I explain the social problematics of childhood obesity and dropping of the Mediterranean diet.			X	X	2.46
16. In my teaching, I include health education to promote healthy eating habits.			X		2.84
17. When dealing with healthy eating habits in the classroom context, these are related with physical activity.			X		2.90
18. The contents on eating habits that I teach are related to the food taken by students during the break.			X	X	2.06
19. The contents on eating habits that I teach are related to the importance of having breakfast at home.			X	X	2.30
20. Regarding eating habits, I only use the content proposed in the textbook.			X		1.64
21. In my lessons, I use the materials designed by certain institutions such as the Ministry of Education or the Regional Ministry of Health and Education.			X	X	1.80
22. When dealing with eating habits, I use the bibliographic resources available at the school library.			X	X	1.22
23. I collect data on students’ eating habits.				X	1.82
24. Regarding eating habits, I only use the activities proposed by the Tutorial Action Plan.				X	2.07
25. I use the Food Guide Pyramid in my lessons.			X	X	2.25
**Teacher training**	**MT**	**COUNSEL.**	**TEACHERS**	**TUTOR**	**Global means**
1. The management team participates in training activities regarding eating habits and health.	X	X			1.74
2. There is a research and innovation line working on health promotion.	X				1.29
3. I collaborate with the school research and innovation lines that work on health promotion.		X	X	X	1.37
4. I develop curriculum material for the development of eating habits content.			X	X	1.96
5. I participate in the training activities designed by the eating habits and nutrition committee.			X	X	1.56
6. I turn to experts to find support for self-training in eating habits and nutrition.			X		1.68
7. I have taken part in some experiences related to the promotion of healthy eating habits in the school environment.			X	X	1.57
8. In case of having taken part in any of these experiences, was it satisfactory?			X	X	2.36
9. I am aware of the indicators that define the centre’s Educational Project regarding the promotion of healthy eating habits.			X		1.75
**Community participation**	**MT**	**COUNSEL.**	**TEACHERS**	**TUTOR**	**Global means**
1. After-school activities are developed in joint action with the community to promote healthy eating habits.	X	X			1.64
2. Actions on healthy eating habits integrated in the centre’s Educational Project include the participation of the Parents’ Association.	X	X			1.59
3. Actions on healthy eating habits integrated in the centre’s Educational Project include the participation of other institutions and/or community services: Health Centres, municipal services, etc.	X	X			2.19
4. Cooking workshops are carried out with community participation and cooperation.	X	X			1.27
5. Documentation on healthy eating habits is provided to families, Parents’ Associations and the school cafeteria staff.	X	X			1.35
**Community training**	**MT**	**COUNSEL.**	**TEACHERS**	**TUTOR**	**Global means**
1. Actions on healthy eating habits are carried out and aimed at the school cafeteria staff.	X	X			1.35

* Informant’s profile: MT, Management Team, COUNSEL., Counsellor, TEACHERS, Teachers instructing in the topic subjects, TUTOR, Course tutor.

## References

[1] González Rodríguez A., Travé González  G., García Padilla F.M. (2019). La mejora de los hábitos de desayuno y merienda escolar a través de una doble intervención escuela familia. REID Revista Electrónica de Investigación y Docencia.

[2] Waters E., de Silva-Sanigorski A., Burford B.J., Brown T., Campbell K.J., Gao Y., Armstrong R., Prosser L., Summerbell C.D. (2011). Cochrane Database of Systematic Reviews Interventions for preventing obesity in children (Review). Cochrane Database Syst. Rev..

[3] Ponce-Ponce de León G., Rieke-Campoy U., Camargo Bravo A., Magaña Rosas A. (2016). Impacto de un programa de promoción de alimentación saludable en el IMC y en los hábitos de alimentación en alumnos de educación secundaria (Impact of a programme for the promotion of healthy eating in the BMI and students in secondary education in eating habits). RICS Revista Iberoamericana de las Ciencias de la Salud.

[4] Pareja Sierra S.L., Roura Carvajal E., Milà-Villarroel R., Adot Caballero A. (2018). Study and promotion of healthy eating habits and physical activity among Spanish adolescents: TAS program (you and Alicia for health). Nutr. Hosp..

[5] Sánchez-Cruz J.J., Jiménez-Moleón J.J., Fernández Quesada F., Sánchez M.J. (2013). Prevalencia de obesidad infantil y juvenil en España. Revista Española de Cardiología.

[6] Rathi N., Riddell L.J., Worsley A. (2018). Urban Indian adolescents practise unhealthy dietary behaviours. Br. Food J..

[7] OMS (WHO) Estrategia Mundial Sobre Régimen Alimentario, Actividad física y Salud. https://www.who.int/dietphysicalactivity/childhood/es/.

[8] WHO (2018). Obesity and Overweight. http://www.who.int/mediacentre/factsheets/fs311/en/.

[9] OMS (WHO) (2015). Alimentación sana. http://www.who.int/mediacentre/factsheets/fs394/es/.

[10] Cofino R., Aviñó D., Benedé C.B., Botello B., Cubillo J., Morgan A., Hernán M. (2016). Promoción de la salud basada en activos: ¿cómo trabajar con esta perspectiva en intervenciones locales?. Gac. Sanit..

[11] Banet E., López C. (2010). ¿Cómo mejorar el desayuno de los escolares de educación primaria?. Revista de Investigación en la Escuela.

[12] Pérez-López I.J., Tercedor-Sánchez P., Delgado-Fernández M. (2015). Efectos de los programas escolares de promoción de actividad física y alimentación en adolescentes españoles: Revisión sistemática. Nutr. Hos..

[13] Rathi N., Riddell L.J., Worsley A. (2016). What influences urban Indian secondary school students’ food consumption? A qualitative study. Appetite.

[14] Garrido-Fernández A., García-Padilla F.M., Sánchez-Ramos J.L., Gómez-Salgado J., Travé-González G.H., Sosa-Cordobés E. (2020). Food consumed by High School Students during the School Day. Nutrients.

[15] Herrero Lozano R., Fillat-Ballesteros J.C. (2010). Influencia de un programa de educación nutricional en la modificación del desayuno en un grupo de adolescentes. Nutrición Clínica y Dietética Hospitalaria.

[16] Alsharairi N.A., Somerset S.M. (2016). Skipping breakfast in early childhood and its associations with maternal and child BMI: A study of 2–5-year-old Australian children. Eur. J. Clin. Nutr..

[17] Aranceta J. (2008). Obesidad infantil: Nuevos hábitos alimentarios y nuevos riesgos para la salud. Análisis de sus causas. Díaz, C. and Gómez, C. (Coord.): Alimentación, consumo y salud. Colección Estudios Sociales.

[18] Jaúregui-Lobera I. (2011). Desayuno y funciones cognitivas en la infancia y la adolescencia. Una revisión sistemática. RENC Revista Española Nutrición Comunitaria.

[19] Serra L., Aranceta J. (2004). Nutrición infantil y juvenil. Estudio enKid.

[20] Adolphus K., Lawton C.L., Champ C.L., Dye L. (2016). The effects of breakfast and breakfast composition on cognition in children and adolescents: A systematic review. Adv. Nutr..

[21] Serra L., Aranceta J. (2004). Desayuno y equilibrio alimentario. Estudio enKid.

[22] Palenzuela Paniagua S.M., Pérez Milena A., Pérula de Torres L.A., Fernández García J.A., Maldonado Alconada J. (2014). La alimentación en el adolescente. An. Sist. Sanit. Navar..

[23] Varela G., Belmonte S., Rivero-Urgell L.A., Moreno J., Dalmau J.M., Moreno A., Aliaga A., García-Perea J.M. (2015). El entorno escolar. El libro blanco de la alimentación escolar.

[24] Rathi N., Riddell L.J., Worsley A. (2017). Food and nutrition education in private Indian secondary schools. Health Educ..

[25] Rathi N., Riddell L.J., Worsley A. (2019). Parents’ and teachers’ critique of nutrition education in Indian secondary schools. Health Educ..

[26] Rathi N., Riddell L.J., Worsley A. (2020). Perceptions of eating and food preparation behaviours for urban private school students in India. Child Adolesc. Obes..

[27] García-Padilla F.M., González Rodríguez A. (2017). Cafeteria services and promoting health in the school environment. Aten Primaria.

[28] González Rodríguez A., García-Padilla F.M., Martos Cerezuela I., Silvano Arranz A., Fernández Lao I. (2015). Project ANDALIES: Consumption, supply and promotion of healthy eating in the secondary education centres of Andalusia. Nutr. Hosp..

[29] Rathi N., Riddell L.J., Worsley A. (2018). Parents’ and Teachers’ Views of Food Environments and Policies in Indian Private Secondary Schools. Int. J. Environ. Res. Public Health.

[30] González-Rodríguez A., Travé-González G.H., García-Padilla F.M. (2020). La educación nutricional a partir del trabajo por proyectos en Educación Primaria. Revista Didáctica de las Ciencias Experimentales y Sociales..

[31] Langford R., Campbell R., Magnus D., Bonell C.P., Murphy S.M., Waters E., Gibbs L.F. (2014). The WHO Health Promoting School framework for improving the health and well-being of students and staff. Cochrane Database Syst. Rev..

[32] Schwartz A.E., Leardo M., Aneja S., Elbel B. (2016). Effect of a school-based water intervention on child body mass index and obesity. JAMA Pediatrics.

[33] Torres-García M., Santana-Hernández H. (2017). La Educación para la Salud en la formación de maestros desde el Espacio Europeo de Educación Superior. Rev. Complut. Educ..

[34] Jones A.M., Zidenberg-Cherr S. (2015). Exploring Nutrition Education Resources and Barriers, and Nutrition Knowledge in Teachers in California. J. Nutr. Educ. Behav..

[35] Dos Santos Rocha A., Barbosa Facina V. (2017). Professores da rede municipal de ensino e o conhecimento sobre o papel da escola na formação dos hábitos alimentares dos escolares. Ciênc. educ. (Bauru).

[36] González-Rodríguez A., Travé-González G., García-Padilla F.M. (2018). Eating habits, physical activity and hours of sleep in school children: A diagnostic study in Primary Education. Educ. Siglo XXI..

[37] Rodrigo-Vega M., Ejeda-Manzanera J.M., Caballero-Armenta M., Cubero-Juánez J., Ortega-Navas C. (2017). Las Guías Alimentarias como material didáctico en la formación de Maestros: Análisis y aplicación. Revista Complutense de Educación.

[38] Black A.P., D’Onise K., McDermott R., Vally H., O’Dea K. (2017). How effective are family-based and institutional nutrition interventions in improving children’s diet and health? A systematic review. BMC Public Health.

[39] García-Padilla F.M., González-Rodríguez A., González-de Haro M.D., Frigolet-Maceras J. (2012). La promoción de la alimentación saludable en Educación Secundaria: Consenso sobre indicadores de valoración (Promoting healthy eating in Secondary Education: Consensus on assessment indicators). Rev. Esp. Nutr. Comunitaria.

[40] BOE 194 Ley Orgánica 3/2018, de 5 de diciembre, de Protección de Datos Personales y garantía de los derechos digitales. https://boe.es/buscar/act.php?id=BOE-A-2018-16673.

[41] Mundial A.M. (2013). Declaración de Helsinki de la AMM-Principios éticos para las investigaciones médicas en seres humanos-WMA-The World Medical Association. https://www.wma.net/es/policies-post/declaracion-de-helsinki-de-la-amm-principios-eticos-para-las-investigaciones-medicas-en-seres-humanos/.

[42] BOE 295 Ley Orgánica 8/2013, de 9 de diciembre, para la mejora de la calidad educativa (LOMCE). https://www.boe.es/buscar/pdf/2013/BOE-A-2013-12886-consolidado.pdf.

[43] Rathi N., Riddell L., Worsley A. (2018). The role of Indian school canteens in nutrition promotion. Br. Food J..

[44] BOE 160 Ley 17/2011, de 5 de julio, de seguridad alimentaria y nutrición. https://www.boe.es/diario_boe/txt.php?id=BOE-A-2011-11604.

[45] BOJA 139 Decreto 327/2010, de 13 de julio, por el que se aprueba el Reglamento Orgánico de los Institutos de Educación Secundaria. https://www.juntadeandalucia.es/boja/2010/139/2.

[46] Sharma M. (2011). Dietary Education in School-Based Childhood Obesity Prevention Programs. Adv. Nutr..

[47] Van Cauwenberghe E., Maes L., Spittaels H., van Lenthe F.J., Brug J., Oppert J.M., De Bourdeaudhuij I. (2010). Effectiveness of school-based interventions in Europe to promote healthy nutrition in children and adolescents: Systematic review of published and ’grey’ literature. Br. J. Nutr..

[48] Garrido-Fernández A., García-Padilla F.M., Sánchez-Ramos J.L., Gómez-Salgado J., Sosa-Cordobés E. (2020). The Family as an Actor in High School Students’ Eating Habits: A Qualitative Research Study. Foods.

[49] Rush E., Cairncross C., Williams M.H., Tseng M., Coppinger T., McLennan S., Latimer K. (2016). Project Energize: Intervention development and 10 years of progress in preventing childhood obesity. BMC Res. Notes.

[50] Garcia-Silva J., Navarrete Navarrete N., Silva-Silva D., Caparros-Gonzalez R.A., Peralta-Ramírez M.I., Caballo V.E. (2019). Escalas de Apoyo Social para los Hábitos Alimentarios y para el Ejercicio: Propiedades Psicométricas. Rev. Española Salud Pública.

